# Age-specific seroprevalence of human polyomavirus 12 and Saint Louis and New Jersey polyomaviruses

**DOI:** 10.1038/s41426-018-0026-0

**Published:** 2018-03-07

**Authors:** Pauline Gaboriaud, Marion Ferté, Françoise Arnold, Valérie Leblond, Jérôme Nicol, Heloïse Debare, Mélanie Le Meur, Fernanda Martini, Mauro Tognon, Antoine Touzé

**Affiliations:** 1 UMR INRA ISP 1282, team Biologie des infections à polyomavirus, Université de Tours, Tours 31, Avenue Monge, 37200 Tours, France; 2 UMR INRA ISP 1282, team BioMédicaments Antiparasitaires, Université de Tours, Tours 31, Avenue Monge, 37200 Tours, France; 3 Department of Morphology, Surgery and Experimental Medicine, Section of Pathology, Oncology and Experimental Biology, Laboratories of Cell Biology and Molecular Genetics, University of Ferrara, 44121 Ferrare, Italy

## Abstract

The presence of specific antibodies against human polyomavirus 12, Saint Louis polyomavirus and New Jersey polyomavirus was investigated by using virus-like particle-based ELISAs with serum samples from 706 Italians aged 1- to 100-years-old. The findings indicate that these polyomaviruses circulate widely in humans, with peak seroprevalence, observed at adulthood, of 97.3%, 93.3%, 57.5%, for human polyomavirus 12, Saint Louis polyomavirus and New Jersey polyomavirus, respectively.

## Introduction

Seven new human polyomaviruses (PyVs) have been identified since the discovery of Merkel cell polyomavirus in 2008^1, 2^. Three recently identified PyVs are human polyomavirus 12 (HPyV12)^3^, Saint Louis polyomavirus (STLPyV)^4^ and New Jersey polyomavirus (NJPyV)^5^. STLPyV, classified as a Delta-polyomavirus^6^, was identified in stools from a healthy Malawian child^4^. Previous studies reported a seroprevalence of 68 to 70% and an early exposure in the United States^7^ as described for other recently discovered PyVs^8, 9^. HPyV12 was classified as an Alpha-polyomavirus and was discovered in resected livers^3^. The same study reported a moderate seroprevalence (15–33%) in a healthy adult population^3^. Finally, NJPyV, classified as an Alpha-polyomavirus, was discovered in a pancreatic transplant recipient presenting retinal blindness and vasculitic myopathy^5^. Little is known about the natural history of these new human PyVs. The aim of this study was to investigate their age-specific seroprevalence in an Italian general population from the Ferrara region by using virus-like particle (VLP)-based ELISAs.

## Materials and methods

### Subjects and samples

Participants (*n* = 706; 416 females) ranged in age from 1- to 100-years-old. Serum samples investigated were part of the collection used in previous studies^8–10^. The County Ethics Committee of Ferrara, Italy, approved the project. Consent from participants was not requested for polyomavirus testing, and samples were, therefore, de-identified and analyzed anonymously, with indication of age and gender only. Samples were stored at − 20°C until testing.

### Production of VLPs

**Fig. 1:**
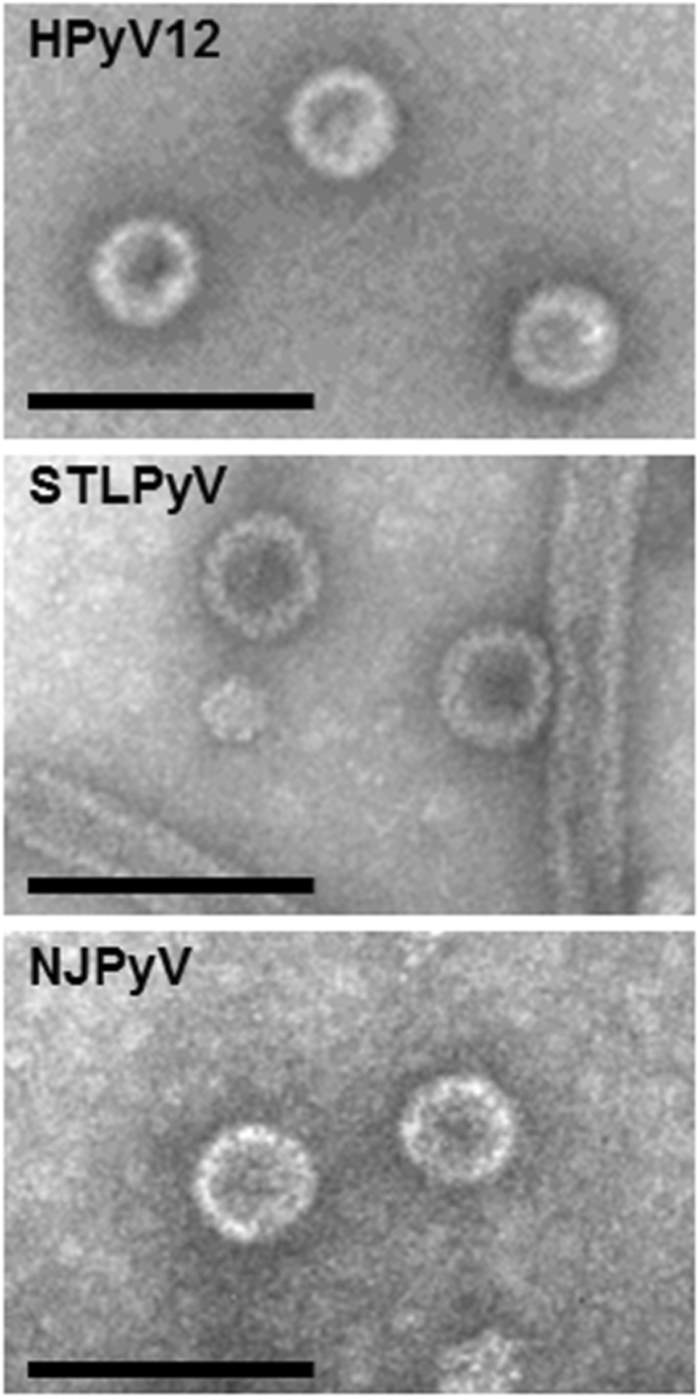
Electron microscopy of HPyV12, STLPyV and NJPyV virus-like particles (VLPs) produced in *Spodoptera frugiperda* cells. The expected size of VLPs is 45–50 nm. Scale bars, 100 nm

VLPs were generated for STLPyV, HPyV12 and NJPyV. Briefly, the VP1 coding sequences (GenBank: STLPyV, KF525270; HPyV12, JX308829.1; NJPyV NC024118) were obtained by synthesis (Genscript, Piscataway, NJ). The different VP1 genes were cloned under the control of the polyhedrin promoter of the pFastBac Dual plasmid and further used to generate recombinant baculoviruses, using the Bac-to-Bac system (Invitrogen, FisherScientific, Illkirch, France). Sf21 cells maintained in Grace medium (Invitrogen) were infected with the different recombinant baculoviruses for production of the three polyomavirus VLPs. VLPs were then purified by ultracentrifugation (18 h at 30,000 rpm in a Beckman SW 32 rotor) in a CsCl gradient and the fraction with a density of 1.272 was diluted in phosphate-buffered saline (PBS) and submitted to ultracentrifugation (3 h at 32,000 rpm in a Beckman SW 32 rotor). The pellet was then resuspended in PBS and the preparations were applied to carbon grids, negatively stained with 1.5% uranyl acetate and observed with a JEOL 1011 electron microscope at 50,000 nominal magnification (Fig. 1).

### Detection of anti-VP1 antibodies

Microplates (Maxisorp; Nunc) were coated overnight at 4 °C with SLTPyV, HPyV12 and NJPyV VLPs (100 ng/well in PBS) as previously described^9–11^. Briefly, sera were diluted 1:100, and peroxidase-conjugated anti-human IgG (Southern Biotech, Clinisciences, Nanterre, France) diluted 1:20,000 was used to detect human IgG binding. By using net optical density (OD) obtained from the 706 samples, a tendency curve was drawn from a second-degree polynomial regression (for STLPyV and HPyV12) or third-degree polynomial regression (for NJPyV) (not shown). These representations revealed inflection points corresponding to cutoff values of 0.200, 0.230 and 0.230 for STLPyV, HPyV12 and NJPyV, respectively. Samples were considered to have high levels of antibodies when the OD value was greater than the median for the seropositive samples (i.e., 0.793 for STLPyV and 0.475 for HPyV12 and 0.481 for NJPyV).

### Statistical methods

Odds ratios (ORs) with 95% confidence intervals (95% CI) were obtained to assess the magnitude and statistical significance of the associations between high levels of antibodies, gender and age. Correlation analysis of polyomavirus seroreactivity involved the Spearman rank correlation test (*R*_S_ value). Statistical analysis involved use of XLStat software (Addinsoft, France).

## Results

To evaluate virus-specific prevalences and to rule out cross-reactivity among HPyV12, STLPyV, NJPyV and other PyVs previously investigated^8–10^, we performed Spearman correlation of OD values (Table 1) and found no strong correlation among the PyVs (all *R*_s_ < 0.4). Nevertheless, we observed a slight correlation between MCPyV and NJPyV (*R*_S_ = 0.357). ELISA competition experiments were performed by preincubating sera with a 20-fold excess amount of soluble heterologous VLPs for these two viruses with serum presenting high-level dual reactivity. Preincubation with heterologous VLPs did not affect reactivity, whereas preincubation with homologous VLPs totally abolished the signals (data not shown).

**Table 1:** Spearman correlation coefficients for seroreactivity in ELISAs between HPyV12, STLPyV, NJPyV and the six human polyomaviruses previously described^9–11^

	RS value^a^ (*P*-value)								
	HPyV12	STLPyV	NJPyV	MCPyV	HPyV6	HPyV7	TSPyV	HPyV9	MWPyV
HPyV12	1	0.330 (<0.0001)	0.230 (<0.0001)	0.049 (0.214)	0.129 (0.001)	0.114 (0.003)	0.013 (0.745)	0.006 (0.886)	0.025 (0.529
STLPyV		1	0.099 (0.012)	0.093 (0.017)	0.125 (0.001)	0.025 (0.525)	0.110 (0.005)	0.073 (0.062)	0.169 (<0.0001)
NJPyV			1	0.357 (<0.0001)	0.158 (<0.0001)	0.126 (0.001)	0.224 (<0.0001)	0.268 (<0.0001)	0.072 (0.065)

^a^RS value, Spearman correlation coefficient

**Fig. 2:**
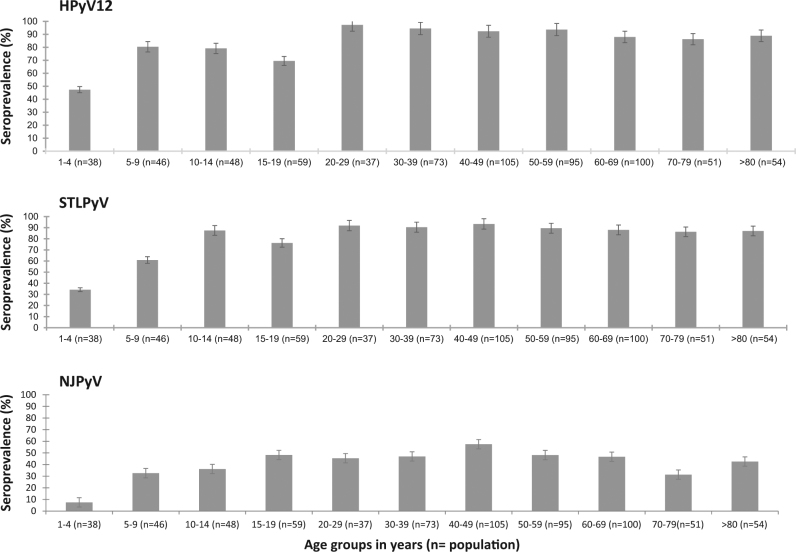
Age-specific seroprevalences of HPyV12, STLPyV and NJPyV. Data are percentage and 95% confidence intervals

Because of no cross-reactivity found, we then investigated age-specific seroprevalences for HPyV12, STLPyV and NJPyV (Fig. 2). For HPyV12 and STLPyV, the seroprevalence increased with age, from 47% and 34% in children 1- to 4-years-old, respectively, to 80% and 61% in those 5- to 9-years-old, respectively. The seroprevalence peaked at 97.3% and 93.3% for HPyV12 and STLPyV (range 20–29 years for both), then plateaued at about 90% for the rest of the life. The seroprevalence of NJPyV clearly differed from that of the two other PyVs, with a seropositivity of only 7.5% in children from 1- to 4-years-old. Children 5- to 9-years-old showed a clear increase in seropositivity, to 33%. Then, the NJPyV seroprevalence increased steadily to 48% in children 15- to 19-years-old and the upper value was observed in subjects 40- to 49-years-old (57.5%). After that, the seroprevalence was stable, with a moderate reduction in individuals 70- to 79-years-old (31.4%).

Distributions of OD values for the three polyomaviruses studied indicated that high values, which can reflect the level of virus replication, were observed for STLPyV but not for HPyV12 and NJPyV (data not shown). Samples were considered to have high levels reactivity when the OD value was greater than the median for the seropositive samples (i.e., 0.793 for STLPyV, 0.475 for HPyV12 and 0.481 for NJPyV). The high reactivity did not differ by sex for the three polyomaviruses (Table 2). The same results were observed in the overall population (data not shown).High OD values for HPyV12 was increased but stable from age 20 to > 80 years, with significant ORs ranging from 2.32 to 2.74 (Table 2), whereas for STLPyV, for participants older than age 9, we observed a significant negative association of high OD values (ORs from 0.23 to 0.39). Concerning NJPyV, the high OD value proportions markedly varied by age range. Only 23% of participants 10- to 19-years-old had high OD values as compared with 72% of those 20- to 39-years-old (OR = 2.87; 95% CI 0.99–8.36, *P* = 0.052).

**Table 2:** High levels of HPyV12, STLPyV and NJPyV in seropositive subjects according to age and sex^a^

Virus	Group	No. of subjects with high level /no. of positive subjects (%)	OR (95% CI)	*P*-value^b^
HPyV12	Females	188/360 (52.2)	1	
	Males	114/245 (46.5)	0.79 (0.57–1.10)	0.169
	1–9	18/55 (32.7)	1	
	10–19	28/79 (35.4)	1.13 (0.54–2.33)	0.744
	20–39	60/105 (57.1)	2.74 (1.38–5.42)	**0.0038**
	40–59	99/186 (53.2)	2.34 (1.24–4.40)	**0.0085**
	60–79	70/132 (53.0)	2.32 (1.20–4.48)	**0.0123**
	>80	27/48 (56.2)	2.64 (1.18–5.89)	**0.0** **175**
STLPyV	Females	172/350 (49.1)	1	
	Males	122/240 (50.8)	1.07 (0.77–1.48)	0.686
	1–9	25/33 (75.7)	1	
	10–19	42/87 (48.2)	0.29 (0.12–0.73)	**0.008**
	20–39	49/100 (49.0)	0.31 (0.12–0.74)	**0.009**
	40–59	101/183 (55.1)	0.39 (0.17–0.92)	**0.031**
	60–79	55/132 (41.6)	0.23 (0.09–0.54)	**<** **10** ^**−3**^
	>80	22/47 (46.8)	0.28 (0.10–0.75)	**0.011**
NJPyV	Females	71/166 (42.8)	1	
	Males	48/103 (46.6)	1.16 (0.71–1.91)	0.538
	1–9	8/18 (44.4)	1	
	10–19	10/44 (22.7)	0.37 (0.11–1.18)	0.093
	20–39	46/64 (71.9)	2.87 (0.99–8.36)	0.052
	40–59	42/89 (47.2)	1.11 (0.40–3.09)	0.831
	60–79	35/59 (59.3)	1.82 (0.62–5.28)	0.269
	>80	15/54 (27.8)	0.48 (0.16–1.45)	0.193

^a^The median ODs for seropositive samples (0.475, 0.793 and 0.481 for HPyV12, STLPyV and NJPyV, respectively) were used as cutoffs for high antibody levels

^b^Significant *P*-values are in bold

## Discussion

As was observed for other PyVs investigated, the three new PyVs did not show cross-reactivity with the previously investigated PyVs, even between MCPyV and NJPyV, for which we observed a moderate correlation of seroreactivity (*R*_s_ = 0.357). Since we did not observe competition on preincubation experiments, this correlation is likely due to co-infection, as evidenced by Gossai et al.^11^. To study further co-infections, we analyzed the seroprevalence data obtained in this work and pooled them with previously published results from the same population^8–10^. Overall, the results confirmed that seroconversions occur during childhood and that by the end of childhood, the population has markers of infection with at least six of the nine PyVs investigated. This finding also corresponds to the overall population rate for PyV seropositivity. Positivity for the nine PyVs was observed after age 60 (data not shown).

Antibodies to STLPyV VP1 were detected in >90% of adults ( > 20 years old), a proportion higher than the overall seropositivity of 70% reported in the United States^7^. This difference in seropositivity is probably due to the structure of the antigen used but may also be due to a real difference in study population characteristics. Primary exposure to STLPyV probably occurred mainly in early childhood, because we observed a seroprevalence of 61% in children 5- to 10-years-old. This seroprevalence was similar to the 61.1 to 70.8% reported for individuals 4- to 20-years-old in the United States^7^. The seroprevalence by age classes for STLPyV is also comparable to that for other PyVs previously investigated^8^ and comparable to that observed for HPyV12. In adulthood, the seroprevalence is >95%. The primary exposure to HPyV12 probably also occurs mainly in early childhood because of a seroprevalence of about 80% observed in children 5- to 9-years-old. Because HPyV12 was first detected in the gastrointestinal tract and particularly in individuals with malignant liver diseases, the evaluation of anti-HPyV12 antibodies titre in such settings may be of interest to try to identify an implication in the aetiology of these cancers^5^. High-level reactivity was negatively associated with age for STLPyV but positively with age for HPyV12. The latter suggestion implies continuous replication of HPyV12. Even though high-level reactivity was determined with a single serum dilution, it was found a good surrogate for high antibody titre^8^.

The level of seroprevalence for NJPyV slightly differed from that for the other PyVs investigated and those previously studied because the higher seroprevalence is only 57% in adults. Nevertheless, such a low level of seroprevalence was previously observed for HPyV9 but for older individuals^8^. The differences in seroprevalence for these three new human PyVs suggest that they may have different transmission modes and capacities for persistence.
